# Physiological correlates of interoception and the effect of heart rate variability biofeedback

**DOI:** 10.3389/fnetp.2026.1846014

**Published:** 2026-07-01

**Authors:** Andy Schumann, Lisa Schmitt, Katrin Rieger, Elif Çalışkan, Feliberto De la Cruz, Maria Geisler, Yubraj Gupta, Karl-Jürgen Bär

**Affiliations:** 1 Lab for Autonomic Neuroscience, Imaging and Cognition (LANIC), Department for Psychosomatic Medicine and Psychotherapy, Jena University Hospital, Jena, Germany; 2 Department for the Psychology of Human Movement and Sport, Institute of Sports Science, Friedrich Schiller University Jena, Jena, Germany; 3 Department of Psychology, Institute of Postgraduate Education, İzmir Tınaztepe University, İzmir, Türkiye

**Keywords:** baroreflex sensitivity, heartbeat perception, interoceptive awareness, network physiology, respiratory sinus arrhythmia

## Abstract

**Introduction:**

Interoception, the perception and interpretation of internal bodily signals, is closely linked to autonomic regulation and emotional functioning. Heart rate variability biofeedback (HRVBF) has been proposed to influence interoceptive processes through modulation of vagally mediated cardiovascular dynamics. The present study investigated physiological correlates of interoception and examined whether an 8-week HRVBF intervention was associated with changes in autonomic regulation and interoceptive processing in healthy adults.

**Methods:**

Twenty-five participants completed an 8-week HRVBF training. Resting autonomic function was assessed before and after the intervention using cardiovascular and respiratory indices, including heart rate, heart rate variability, baroreflex sensitivity, respiratory sinus arrhythmia (RSA), respiration rate, and spectral HRV measures. Interoceptive accuracy was assessed using the heartbeat counting task, and interoceptive awareness was assessed using the Multidimensional Assessment of Interoceptive Awareness (MAIA).

**Results:**

At baseline, lower respiration rate and heart rate were associated with higher scores on several MAIA subscales. The HRVBF intervention was associated with significant multivariate changes in autonomic function, including decreased respiration rate, increased RSA, and elevated baroreflex sensitivity. Interoceptive accuracy improved modestly following the intervention. Interoceptive awareness also showed a significant multivariate change, with largest increases observed in the subscale Self-Regulation.

**Conclusion:**

In conclusion, HRVBF was associated with modulation of resting autonomic regulation and improvements in selected dimensions of interoceptive processing, particularly subjective self-regulatory awareness. Associations between physiological and interoceptive changes were modest, suggesting further research is needed to clarify the relationship between autonomic regulation and interoceptive function.

## Introduction

The roots of interoception can be traced back to ancient theories of mind and body, in which the relationship between bodily sensation and emotions was a central concern. The philosophical foundation for this view begins with Aristotle (350 BCE), who believed that emotions are fundamentally connected to bodily changes, remarking that states such as anger, fear, and pity are always expressed through corresponding bodily responses (Brandt et al., 2024). In contemporary terms, what is now called *interoception *is defined as the perception and interpretation of the body signals, such as heartbeat, respiration, or visceral sensations reflect these early philosophical insights. Modern neuroscience further emphasizes that interoception is not a passive mechanism, but rather a dynamic process that arises from the complex interplay between physiological sensitivity and cognitive interpretation (Craig, 2002; Critchley and Garfinkel, 2017).

The detection of visceral signals and the meaning attributed to them jointly shape emotional experience, behavioral responses, and overall wellbeing. While interoception includes a wide variety of visceral modalities, the cardiovascular pathway is the most extensively investigated in cognitive neuroscience, due to its practical measurability and strong link to affective experiences (Fittipaldi et al., 2020; Garfinkel et al., 2015; Garfinkel and Critchley, 2016). The ability to process internal cardiac signals, known specifically as *cardiac interoception*, is considered a crucial mechanism in autonomic control and emotion regulation. This process typically consists of three measurable dimensions: *interoceptive accuracy* (the objective detection of signals, often measured via the heartbeat counting task), *interoceptive sensibility* (self-reported beliefs about one’s ability), and *interoceptive awareness* (the correspondence between the former two dimensions) (Garfinkel et al., 2015). Emerging evidence demonstrates that a heightened capacity to detect and process the internal bodily sensations provides a significant physiological foundation for adaptive self-regulation, particularly under stress (Critchley and Garfinkel, 2017; Schulz and Vögele, 2015).

The vagus nerve of the parasympathetic autonomic nervous system serves as the primary afferent pathway for transmitting homeostatic information from internal organs to the brain. Several studies report positive associations between vagally mediated heart rate variability (HRV) and interoceptive accuracy (heartbeat-detection tasks) and related behavioral measures, suggesting that higher baseline vagal tone is linked to better cardiac interoceptive performance (Lischke et al., 2021).

Breathing is a particularly powerful modulator of interoceptive processes due to its close relationship with vagal function and cardiovascular regulation. A key manifestation of this interaction is respiratory sinus arrhythmia (RSA), the rhythmic fluctuation of heart rate across the respiratory cycle. During inhalation, vagal activity is transiently inhibited, leading to an acceleration of heart rate, while exhalation restores vagal influence, resulting in a deceleration (Berntson et al., 1993). Slow, paced breathing influences heart rate variability through the baroreflex, a homeostatic mechanism that adjusts heart rate in response to changes in blood pressure (Vaschillo et al., 2006). This reflex arc involves mechanosensitive receptors in the aortic arch and carotid sinus, which send afferent signals via the vagus and glossopharyngeal nerves to the brainstem, eliciting compensatory adjustments in cardiac vagal output (Benarroch, 1993).

During heart rate variability biofeedback (HRVBF), participants train to increase HRV primarily by modifying their breathing pattern, typically through slow, deep, diaphragmatic breathing. HRV is visually fed back to participants in real time (e.g., via graphs, animations, or coherence scores), allowing them to learn how to adjust their breathing and attention to enhance physiological regulation.

Multiple systematic reviews and meta-analyses converge on a modest-to-moderate beneficial effect of HRVBF on self-reported stress and anxiety. For example, Goessl and colleagues (2017) found large reductions in self-reported anxiety across trials, and larger recent meta-analytic reports show small–moderate effects across emotional and some physical outcomes (Lehrer et al., 2020; Pizzoli et al., 2021). These syntheses support the claim that HRVBF is an effective psychophysiological intervention for a range of outcomes, but effect sizes and certainty vary by outcome and population.

Mechanistic accounts of HRVBF integrate autonomic, brainstem, and cortical pathways. Paced breathing at or near an individual’s resonance frequency amplifies RSA and engages the baroreceptor reflex, producing large, ordered oscillations in heart rate. Repeated practice of slow breathing has also been shown to exert lasting effects on resting autonomic function (Schumann et al., 2019). Afferent signals generated by these oscillations travel via the nucleus tractus solitarii (NTS) and vagal pathways, influencing higher-order cortical regions implicated in interoception and emotion regulation, including the insula, anterior cingulate cortex, medial prefrontal cortex, and amygdala. This neurovisceral, or polyvagal, framework provides a plausible pathway by which HRVBF may produce psychological effects (Lehrer and Gevirtz, 2014). Several studies report HRVBF-related changes in functional connectivity, increases in vagal indices during training (Nashiro et al., 2022; Schumann et al., 2021).

Preliminary evidence suggests that HRV biofeedback may also modulate cortical markers of interoceptive processing, such as heartbeat-evoked potentials (HEPs), which are considered an index of vagal afferent–driven cortical activity (Park and Blanke, 2019; Pollatos and Schandry, 2004). In a randomized pilot study, HRVBF training was associated with increased baseline HEP amplitude relative to a relaxation control condition, providing initial support for the hypothesis that HRVBF influences vagal afferent signaling and its cortical representation (Huang et al., 2018).

Several studies have indicated that HRVBF may improve interoceptive processing, although effects are not uniform and appear contingent on adherence to resonance-frequency breathing and sufficient intervention intensity. Interoception refers to the sensing, interpretation, and integration of internal bodily signals, and it has been hypothesized that HRVBF enhances interoception by increasing vagal afferent signaling to the brain via enhanced respiratory sinus arrhythmia and baroreflex sensitivity at resonance frequency, thereby sharpening detection of cardiovascular signals such as heartbeat awareness (Saito et al., 2025).

A recent narrative systematic review of 77 HRVBF studies observed mixed evidence for interoceptive improvements overall (Wareing et al., 2024), but identified trends suggesting that effects on physiological and neural indices related to interoception were more likely when resonance frequency breathing protocols were consistently applied and when interventions were sufficiently intensive (e.g., multiple sessions over weeks). The authors proposed a three-stage model linking HRVBF to improvements in interoceptive processing. In the first stage, resonance-frequency breathing amplifies cardiac and respiratory rhythms, increasing vagal afferent signaling and heart rate variability, thereby strengthening the input to the central nervous system. In the second stage, these signals are processed by the central autonomic network, including the NTS, insula, anterior cingulate cortex, and medial prefrontal cortex, enhancing connectivity and integration of interoceptive information. In the final stage, improved central processing facilitates conscious recognition and interpretation of bodily signals, leading to greater interoceptive accuracy and awareness, which supports adaptive emotion regulation and behavior.

In the current study, we investigated whether an 8-week HRVBF intervention improves interoception in healthy adults. We hypothesized that participants would show increases in both interoceptive accuracy and interoceptive awareness following the biofeedback training. Additionally, we examined whether changes in interoceptive processing were proportional to biofeedback-induced physiological changes at rest, particularly in HRV and related indices of autonomic regulation.

## Methods

### Study group formation

A total of 33 participants were initially enrolled in the study in order to complete an 8-week heart rate variability biofeedback (HRVBF) intervention. During data processing, two participants were excluded due to incomplete baseline data, resulting in a cross-sectional sample of 31 participants (27 female, 4 male) prior to the intervention. Following study withdrawal or the emergence of exclusion criteria during the intervention period, the final longitudinal sample comprised 25 participants (21 female, 4 male) who completed all required sessions. Participants ranged in age from 23 to 60 years (M = 48, SD = 8.7).

Prior to participation, all individuals received written information about the study aims and procedures and provided written informed consent. Participants were informed that they could withdraw from the study at any time without providing a reason and without negative consequences. Upon completion of all study procedures, participants received financial compensation of €80 via the University Hospital Jena research program. Participants were recruited through an online advertisement from the local community. Inclusion criteria comprised an age between 18 and 60 years. Exclusion criteria were: neurological disorders, psychiatric disorders, cardiovascular disease, untreated hypertension, infectious diseases, asthma, cancer, pregnancy, substance abuse, and current use of analgesics or steroids.

### Intervention protocol

The intervention took 8 weeks during which participants in the biofeedback group performed an HRV biofeedback training. Five training sessions per week had to be conducted at home. Heart rate was recorded using a chest-worn sensor (H10 Heart Rate Sensor; Polar Electro Oy, Kempele, Finland). Data were transmitted via Bluetooth to the EliteHRV application (Elite HRV LLC). Heart rate was presented on participants’ smartphones as instantaneous visual feedback. Participants were instructed to enhance heart rate oscillations by breathing abdominally with a slow, deep, and smooth rhythm. After each session, participants emailed their heart rate recordings to the researchers.

**FIGURE 1:**
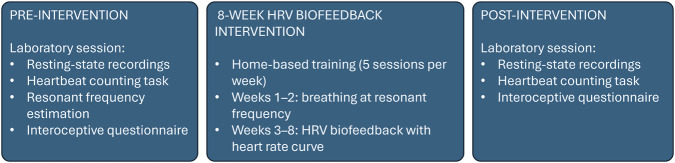
Schematic overview of the study design. Participants completed two laboratory assessment sessions before (Pre) and after (Post) the 8-week HRV biofeedback intervention. Each session included a resting-state recording followed by the heartbeat counting task. At the pre-intervention session, individual resonant frequency was estimated. The intervention consisted of home-based training conducted five times per week.

Participants completed two laboratory assessment sessions: one prior to (baseline/pre-intervention) and one immediately after the 8-week HRV biofeedback intervention (post-intervention). Each laboratory session were scheduled at a similar time of day and consisted of two major components: (1) a resting-state recording and (2) the heartbeat detection task. At pre-intervention, we also assessed resonant frequency (see below). No intermediate assessments were performed (see Figure 1).

### Estimation of resonant frequency

At the pre-intervention session after the resting state recording and heart beat counting task, we estimated each participant’s individual resonant frequency (RF), defined as the breathing rate that maximizes HRV (Lehrer et al., 2000). RF was assessed in the laboratory with participants in the supine position following a resting period. A moving bar displayed on a computer screen guided participants to breathe at rates of 7, 6, 5, 4.5, and 4 breaths per minute for 2 minutes each. During this procedure, electrocardiogram (ECG) signals and respiratory excursions of the chest and abdomen were recorded. The individual RF was identified as the breathing rate associated with the highest HRV, operationalized as the standard deviation of interbeat intervals.

### HRV biofeedback

Biofeedback sessions were performed at home and were self-scheduled by the participants. During the first 2 weeks of training, participants practiced breathing at their RF as displayed in the app to become accustomed to the slow rhythm. Thereafter, they were free to adjust their breathing to maximize heart rate oscillations.

Beginning in the third week, participants’ current HR was displayed on their smartphones as a smoothed, interpolated curve. Participants were instructed to breathe “in phase” with this curve by inhaling during heart rate increases and exhaling during decreases. The primary goal of training was to increase the amplitude of heart rate oscillations, thereby enhancing HRV.

### Assessment of autonomic function

During both pre- and post-intervention laboratory sessions, resting state recordings of ECG, noninvasive blood pressure, and respiration were conducted in supine position for 15 min. The examination room was quiet and fully shaded with a low intensity ambient light source. Via a monitor fixed over the couch a dark gray ellipse was displayed on a light gray background as a fixation anchor. Room temperature was controlled to 22 °C. The first 5 minutes were not analyzed, to allow participants to adjust to the environment.

### Data acquisition and preprocessing

We used the MP150 system (BIOPAC Systems Inc., Goleta, CA, United States) to record multiple physiological signals simultaneously at 1,000 Hz sampling rate. ECG was acquired by three electrodes attached to the chest according to an adjusted Einthoven triangle. Noninvasive blood pressure was measured continuously by the vascular unloading technique (CNAP 500, CNSystems Medizintechnik AG, Graz, AUS). ECG and blood pressure were band-pass filtered between 0.05 and 35 Hz. Abdominal and thoracic respiratory movement were recorded by two individual strain gauge transducers and low pass filtered at 10 Hz. Skin conductance was measured using the constant-voltage technique, with electrodes placed on the middle and ring fingers of the left hand for exploratory purposes but is not included in the present analyses.

### Indices of autonomic function

R-waves were extracted automatically from the ECG and verified manually offline. Artifacts and ectopic beats were detected and interpolated using an adaptive filtering technique (Wessel et al., 2000). Diastolic and systolic blood pressure values within each cardiac cycle were extracted.

Mean HR, global HRV (standard deviation of heart beat intervals, SDNN), vagal short-term HRV (root mean of squared differences of successive heart beat intervals, RMSSD) and spectral power of HRV were estimated in low frequency (0.04–0.15 Hz) and high frequency (0.15–0.5 Hz) and log-transformed using the natural logarithm (Task Force, 1996). For spectral analysis, the heart beat interval time series was interpolated and resampled at 4 Hz to obtain an evenly spaced signal. Additionally, respiratory sinus arrhythmia was calculated using the peak-valley-approach (Grossman, 1992). Mean values of systolic and diastolic blood pressure and breathing rate were assessed. Baroreflex sensitivity was estimated by the sequence method quantifying bradycardic changes of heart rate due to blood pressure increases (Malberg et al., 1999).

### Heartbeat detection task

Interoceptive accuracy was assessed during both pre- and post-intervention sessions using the heartbeat detection task according to Schandry, (1981). Participants were instructed to silently count their own heartbeats without physically checking their pulse or engaging in other bodily manipulations. During the task, participants completed six trials of different durations (20 s, 30 s, and 40 s, repeated two times respectively), presented in pseudo-randomized order, with brief rest intervals between trials.

The task was conducted in the supine position immediately after the resting-state recording. To avoid artificial amplification of pulsatory sensations, all sensors and devices that could enhance heartbeat perception were removed before the task, such as the blood pressure cuff. Counting intervals were indicated by visual cues presented on a screen, and a short test run was conducted beforehand to ensure that participants fully understood the instructions. Simultaneously, ECG data were recorded to determine the actual number of heartbeats occurring within each interval. Interoceptive accuracy (IAc) was calculated for each participant using the standard Schandry formula, yielding a value between 0 and 1, with higher scores indicating greater interoceptive accuracy.

### Statistical analysis

All variables were examined for normality using the Kolmogorov–Smirnov test. In addition to HRV spectral power measures, SDNN and respiratory sinus arrhythmia were log-transformed to achieve normal distributions.

To assess the effect of time (pre-vs. post-intervention), a multivariate analysis of variance (MANOVA) with repeated measures was conducted. Significant multivariate effects were followed by paired-samples t-tests to examine pre–post differences in individual variables.

Bivariate linear associations between interoceptive measures and autonomic function at baseline (prior to HRV biofeedback) as well as associations between their pre–post changes were assessed using partial correlation coefficients with corresponding p-values corrected for age. Specifically, heartbeat counting task (HBCT) performance, self-reported interoceptive awareness (MAIA subscales), and autonomic indices were included in these analyses. Statistical thresholds for t-tests and correlation analyses were adjusted for multiple comparisons using the false discovery rate (FDR) procedure according to Benjamini and Hochberg, (1995). The significance level was set at α = 0.05.

## Results

### Physiological correlates of interoception

**FIGURE 2:**
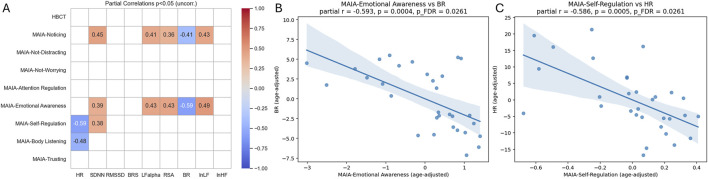
Age-adjusted associations between physiological indices and interoceptive measures, including accuracy in the heartbeat counting task (HBCT) and subscales of the Multidimensional Assessment of Interoceptive Awareness (MAIA). **(A)** Heatmap of uncorrected partial correlation coefficients controlling for age. **(B,C)**. Scatter plots illustrating the relationships between breathing rate (BR, B) and heart rate (HR, C) and MAIA subscale scores after adjusting for age. Axes represent residuals derived from linear regression models including age, such that values reflect deviations from age-predicted levels. Solid lines indicate least-squares regression fits, with shaded areas representing 95% confidence intervals. Abbreviations: HR heart rate; SDNN standard deviation of heart beat intervals; RMSSD root mean squared of successive heart beat interval differences; BRS baroreflex sensitivity; LFalpha baroreflex gain in low frequency range; RSA respiratory sinus arrhythmia; BR breathing rate; lnLF low frequency power, lnHF high frequency power.

We assessed the relationship between markers of interoceptive processing and resting autonomic function using bivariate partial correlations corrected for age (Figure 2). At baseline, prior to the intervention, the MAIA subscale *Noticing* was positively correlated with SDNN (r = 0.45, p = 0.010), LFalpha (r = 0.41, p = 0.022), RSA (r = 0.36, p = 0.048) and lnLF (r = 0.41, p = 0.016), and negatively correlated with BR (r = −0.41, p = 0.022). Similarly, *Emotional Awareness was* positively correlated with SDNN (r = 0.39, p = 0.032), LFalpha (r = 0.43, p = 0.016), RSA (r = 0.43, p = 0.015) and lnLF (r = 0.49, p = 0.005), while negatively correlated with BR (r = −0.59, p < 0.001). *Self-Regulation* was correlated with HR (r = −0.59, p < 0.001) and SDNN (r = 0.38, p = 0.035), while *Body Listening* was proportional to HR (r = −0.48, p = 0.006). Two correlations survived correction for multiple comparisons (Figures 2B,C).

### Effect of HRV biofeedback on resting autonomic function

A multivariate repeated-measures analysis revealed a significant effect of time (pre-to post-intervention) on cardiovascular and respiratory indices. The Hotelling’s T^2^ test indicated a significant multivariate change across the nine physiological variables from baseline to post-intervention (T^2^ = 35.02, F (9, 16) = 2.59, p = 0.046).

**FIGURE 3:**
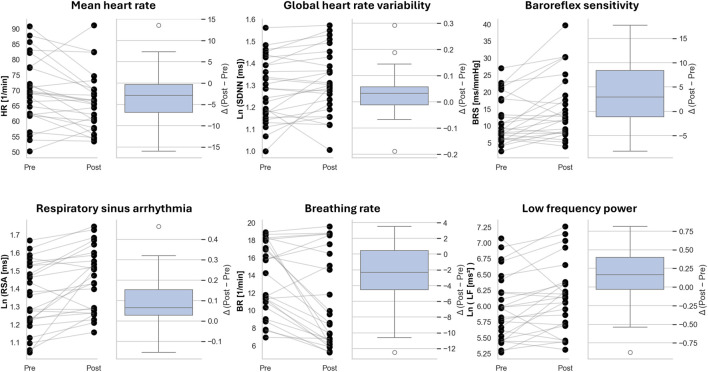
Follow-up paired-samples t-tests comparing autonomic indices at baseline (Pre) and after the biofeedback intervention (Post). Abbreviations: HR heart rate; SDNN standard deviation of heart beat intervals; BRS baroreflex sensitivity; RSA respiratory sinus arrhythmia; BR breathing rate; LF log-transformed low frequency power of heart rate variability, Ln log‐transformed measure.

Follow-up univariate analyses with FDR correction revealed significant pre–post changes in several physiological indices (Table 1; Figure 3). Mean heart rate decreased (d = −0.48, p = 0.025), SDNN increased (d = 0.54, p = 0.034) and lnLF increased (d = 0.42, p = 0.048). But these effects did not remain significant after FDR correction.

**TABLE 1:** Follow-up paired-samples t-tests comparing autonomic indices at baseline (Pre) and after the biofeedback intervention (Post). Correction for multiple comparisons was performed using FDR. Cohen’s *d* effect sizes were calculated based on the mean and standard deviation (SD) of the Pre and Post assessments.

Variable	Pre Mean ± SD	Post Mean ± SD	p-value	Cohen’s d	Corrected p-value
HR [1/min]	69.57 ± 10.74	66.2 ± 9.41	0.025	−0.48	0.0559
SDNN [ms]	43.39 ± 24.37	50.3 ± 29.73	0.034	0.54	0.0607
RMSSD [ms]	33.31 ± 26.88	36.67 ± 23.42	0.322	0.20	0.3623
BRS [ms/mmHg]	11.52 ± 6.88	15.36 ± 9.44	0.004	0.63	0.0129
LFalpha [ms/mmHg]	9.21 ± 5.3	10.51 ± 6.45	0.084	0.36	0.1083
RSA [ms]	66.44 ± 49.04	100.88 ± 80.33	0.001	0.75	0.0055
BR [1/min]	13.75 ± 4.08	11.03 ± 5.32	0.002	−0.69	0.0097
lnLF [ln (ms^2^)]	5.97 ± 0.52	6.12 ± 0.56	0.048	0.42	0.0725
lnHF [ln (ms^2^)]	5.73 ± 0.58	5.77 ± 0.50	0.671	0.09	0.6710

Abbreviations: HR, heart rate; SDNN, standard deviation of heart beat intervals; RMSSD, root mean squared of successive heart beat interval differences; BRS, baroreflex sensitivity; LFalpha baroreflex gain in low frequency range; RSA, respiratory sinus arrhythmia; BR, breathing rate; lnLF, natural logarithm of low frequency power, lnHF, natural logarithm of high frequency power.

Significant increases were observed for BRS (d = 0.63, p = 0.004, p_FDR_ = 0.013) and RSA (d = 0.75, p = 0.001, p_FDR_ = 0.006). Breathing rate decreased significantly (d = −0.69, p = 0.002, p_FDR_ = 0.01). No significant changes were found for RMSSD, LFalpha, or Ln HF.

### Effect of biofeedback on interoceptive accuracy

Interoceptive accuracy, as assessed by the HBCT, was significantly higher following the intervention compared to baseline. A paired-samples t-test revealed an increase from 43% ± 23% at pre-intervention to 51% ± 24% at post-intervention (Cohen’s d = 0.39, p = 0.032), indicating a modest improvement in interoceptive accuracy following the intervention.

### Effect of biofeedback on interoceptive awareness

A multivariate analysis was conducted to examine the effect of time on interoceptive awareness across the eight MAIA subscales. Hotelling’s T^2^ indicated a significant multivariate effect of time, T^2^ = 48.926, corresponding to F (8, 18) = 4.403, p = 0.004, in a sample of 25 participants. These results demonstrate that, taken together, scores on the eight MAIA subscales differed significantly across time points, indicating a reliable overall change in interoceptive awareness over time.

**FIGURE 4:**
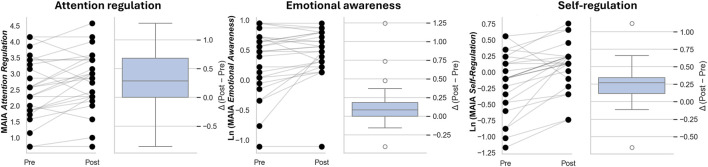
Follow-up paired-samples t-tests comparing subscales of Multivariate Assessment of Interoceptive Awareness (MAIA) at baseline (Pre) and after the biofeedback intervention (Post). Scores on *Emotional Awareness* and *Self-Regulation* have been log-transformed.

Follow-up paired-samples t-tests revealed increases in three MAIA subscales from pre-to post-intervention (Figure 4, Table 2): *Attention Regulation* (p = 0.019, Cohen’s d = 0.49), *Emotional Awareness* (p = 0.040, d = 0.44), and *Self-Regulation* (p < 0.001, d = 0.83). After correction for multiple comparisons using the false discovery rate (FDR) procedure, only the increase in *Self-Regulation* remained statistically significant (corrected p = 0.003).

**TABLE 2:** Follow-up paired-samples t-tests comparing Multidimensional Assessment of Interoceptive Awareness (MAIA) subscales before (Pre) and after the biofeedback intervention (Post). Correction for multiple comparisons was performed using FDR. Cohen’s *d* effect sizes were calculated based on the mean and standard deviation (SD) of the Pre and Post assessments.

MAIA scale	Pre	Post	p-value	Cohen’s d	Corrected p-value
Noticing	3.21 ± 0.99	3.28 ± 0.90	0.6929	0.078	0.7919
Not-distracting	2.12 ± 0.88	2.09 ± 0.81	0.8286	−0.043	0.8286
Not-worrying	2.31 ± 0.98	2.42 ± 1.10	0.5368	0.123	0.7919
Attention regulation	2.53 ± 0.97	2.81 ± 0.91	0.0189	0.492	0.0757
Emotional awareness	3.51 ± 1.18	3.81 ± 0.90	0.0396	0.439	0.1056
Self-regulation	2.43 ± 0.94	2.99 ± 0.79	0.0004	0.830	0.0030
Body listening	2.14 ± 1.12	2.38 ± 1.01	0.2177	0.248	0.4355
Trusting	3.5 ± 1.19	3.59 ± 1.08	0.6239	0.097	0.7919

When examining pre–post changes, positive age-adjusted partial correlations were observed between changes in the MAIA subscale *Not-distracting* and changes in SDNN (r = 0.568, p = 0.003), RSA (r = 0.489, p = 0.013) and lnLF (r = 0.415, p = 0.039). No significant associations were found between changes in HBCT performance and any of the autonomic indices assessed. However, none of these correlations remained significant after FDR correction for multiple comparisons.

## Discussion

The present study examined physiological correlates of interoception and the effects of an 8-week HRVBF intervention in healthy adults. Specifically, we assessed cross-sectional associations between resting autonomic indices and interoceptive accuracy and awareness, as well as whether HRVBF-related changes in autonomic function were accompanied by changes in these interoceptive processing. At baseline, lower resting heart rate and breathing rate were associated with higher emotional awareness and self-regulation. Following the intervention, HRVBF was accompanied by changes in autonomic indices and increases in interoceptive accuracy and awareness.

Interoception is increasingly recognized as a fundamental mechanism supporting emotional experience, self-regulation, and adaptive behavior. Clarifying its physiological underpinnings is therefore essential for understanding how bodily states shape subjective awareness and psychological functioning (Greenwood and Garfinkel, 2025). Reflecting this psychophysiological link, our baseline results showed that lower respiration rate and heart rate were associated with higher scores on different MAIA subscales. This suggests that reduced autonomic arousal may facilitate subjective access to bodily signals, aligning with previous work linking higher vagally mediated HRV to better interoceptive performance (Critchley and Garfinkel, 2017; Lischke et al., 2021).

Consistent with mechanistic models of resonance-frequency breathing, HRVBF induced robust alterations in autonomic indices. Resting heart rate and respiration rate decreased, while BRS and RSA increased, with moderate to large effect sizes. Importantly, increases in BRS and RSA, as well as the reduction in breathing rate, remained significant after correction for multiple comparisons, whereas changes in heart rate, SDNN, and low-frequency HRV did not.

These findings suggest enhanced parasympathetic regulation and strengthened baroreflex function following HRVBF. The observed increase in RSA and baroreflex sensitivity, in particular, supports the notion that repeated training at resonance frequency amplifies vagal afferent signaling and improves autonomic flexibility (Lehrer et al., 2020; Lehrer and Gevirtz, 2014).

The observed increase in low-frequency power is consistent with enhanced baroreflex-mediated oscillatory activity rather than increased sympathetic activity (Moak et al., 2007; Rahman et al., 2011). While RSA is widely used as a proxy for parasympathetic activity, accumulating evidence suggests that it reflects a more complex interaction of respiratory, cardiovascular, and central regulatory processes (Grossman, 2024; Menuet et al., 2025). Recent expert evaluations have further questioned simplified interpretations of RSA and related theoretical frameworks, emphasizing that RSA amplitude does not provide a direct or specific measure of vagal outflow (Grossman et al., 2026). Accordingly, the increases in RSA observed following HRVBF in the present study may reflect enhanced cardiorespiratory coupling and regulatory efficiency rather than isolated changes in vagal activity. Overall, these findings confirm that the intervention successfully targeted the intended physiological mechanisms and align with previous work demonstrating reliable physiological modulation through HRVBF (Vann-Adibe et al., 2025).

Interoceptive accuracy, as assessed by the HBCT, increased modestly following the intervention. This result provides partial support for the hypothesis that amplifying cardiovascular oscillations through resonance breathing enhances access to cardiac afferent signals. Repeated exposure to pronounced heart rate fluctuations during training may facilitate perceptual attunement to internal cardiac cues (Wareing et al., 2024).

However, the effect size was small to moderate, and no significant associations emerged between HBCT performance and resting autonomic indices. This lack of coupling suggests that cardiac interoceptive accuracy may not be proportional to baseline vagal tone or RSA. Alternatively, it may reflect methodological limitations of the HBCT, which is influenced by cognitive estimation strategies and prior beliefs about heart rate (Ring et al., 2018). Thus, while our findings indicate improved task performance, they do not conclusively demonstrate enhanced sensory precision at the afferent level (Brener and Ring, 2016; Körmendi and Ferentzi, 2024).

A multivariate analysis revealed significant overall changes across MAIA subscales, indicating that HRVBF influenced subjective interoceptive awareness. Follow-up analyses showed increases in Attention Regulation, Emotional Awareness, and Self-Regulation. The large effect observed for Self-Regulation suggests that HRVBF primarily enhances individuals’ perceived ability to modulate internal states rather than solely improving perceptual detection of bodily signals.

The ability to regulate distress by attending to bodily signals represents a core component of adaptive emotion regulation, which is closely linked to vagally mediated autonomic flexibility (Craig, 2002; Critchley and Garfinkel, 2017). Thus, improvements in interoceptive self-regulation observed in the present study may reflect a psychological mechanism through which HRVBF exerts its beneficial effects on emotional functioning (Goessl et al., 2017; Pizzoli et al., 2021). HRVBF explicitly trains individuals to observe, regulate, and intentionally influence their physiological states through breathing. Participants repeatedly experience that specific breathing patterns produce predictable interoceptive effects, such as changes in heart rate variability and subjective calmness. Over time, this contingency may strengthen action–effect coupling between respiratory behavior and visceral outcomes, thereby enhancing perceived bodily controllability (Verschooren et al., 2026). As such, improvements in regulatory confidence and perceived bodily control may represent a central mechanism of action. The findings align with neurovisceral integration models, which posit that enhanced vagal regulation is linked to top-down regulatory capacity via medial prefrontal and anterior cingulate pathways (Thayer and Lane, 2000).

Taken together, our findings provide partial support for mechanistic accounts linking HRVBF to enhanced interoceptive processing, as proposed in the staged model by Wareing et al. (2024). The physiological results support Stage 1 of this framework, demonstrating amplified cardiovascular oscillations following training. Improvements in subjective regulatory awareness are consistent with Stage 3, reflecting enhanced conscious interpretation and utilization of bodily signals. However, the present study did not directly assess neural intermediaries within the central autonomic network (e.g., insula, anterior cingulate cortex, medial prefrontal cortex) and therefore cannot directly evaluate Stage 2, which posits enhanced central integration of interoceptive signals.

When examining pre–post associations, increases in the MAIA subscale *Not-Distracting* were positively correlated with changes in SDNN, RSA and lnLF, although these relationships did not survive correction for multiple comparisons. This pattern tentatively aligns with the staged model, suggesting that amplified vagal oscillations (Stage 1) may facilitate improved central processing (Stage 2), ultimately supporting greater conscious engagement with bodily sensations (Stage 3).

Notably, the more pronounced effects on regulatory awareness compared to perceptual accuracy suggest that HRVBF may primarily influence meta-cognitive and regulatory dimensions of interoception rather than raw sensory discrimination. This distinction may be critical for understanding the intervention’s psychological benefits, as enhanced perceived bodily self-regulation may represent a more functionally relevant mechanism than changes in detection accuracy alone.

## Limitations

Several limitations should be considered. First, the absence of a control group precludes definitive causal attribution of observed changes to the intervention. Second, the use of the HBCT as the sole objective interoceptive measure may restrict interpretability due to its known susceptibility to cognitive biases. Finally, the sample size was modest, limiting statistical power, particularly for correlation analyses subjected to multiple comparison correction.

## Conclusion and future directions

In summary, participation in an 8-week HRV biofeedback intervention was associated with changes in resting autonomic regulation and improvements in selected dimensions of interoceptive processing, particularly subjective self-regulatory awareness. While indices reflecting baroreflex and cardiorespiratory dynamics showed reliable modulation over time, the coupling between autonomic and interoceptive changes was modest and requires further investigation.

Future research should use larger randomized controlled designs and include multimodal interoceptive asses sments, such as heartbeat discrimination tasks and neural markers (e.g., heartbeat-evoked potentials or functional neuroimaging), to more directly test the proposed neurovisceral pathway linking HRVBF to interoceptive enhancement. Such work will help clarify whether improvements in autonomic flexibility causally mediate changes in interoceptive capacity and emotional regulation.

## Data Availability

The datasets presented in this article are not readily available because of data protection and privacy regulations. Requests to access the datasets should be directed to Andy Schumann, andy.schumann@med.uni-jena.de.
